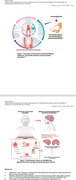# Exploring Neurological and Indirect Biomarkers for Potential Correlations with Oral Health: A Perspective on Dementia Care Practice

**DOI:** 10.1002/alz70858_103578

**Published:** 2025-12-26

**Authors:** Steffany Chamut, Denisse Chamut, Saeideh Dadras, Sondos B. Alghamdi, Bruce A. Dye

**Affiliations:** ^1^ University of Colorado Anschutz Medical Campus, School of Dental Medicine, Aurora, CO, USA; ^2^ Next S‐Miles, San Antonio, TX, USA; ^3^ Colorado School of Public Health (University of Colorado Anschutz Medical Campus), Aurora, CO, USA; ^4^ Harvard School of Dental Medicine, Boston, MA, USA; ^5^ Department of Pediatric Dentisry and Orthodontics, College of Dentistry, King Khalid University, Abha, Aseer Province, Saudi Arabia; ^6^ School of Dental Medicine. University of Colorado Anschutz Medical Campus, Aurora, CO, USA

## Abstract

**Background:**

Poor oral health (OH), including periodontal disease and tooth loss, has been linked to systemic inflammation, neurodegeneration, and cognitive decline, underscoring the critical yet underexplored connection between oral and brain health. Alzheimer's Disease and Related Dementias (AD/ADRD) disproportionately affect aging populations, and with cases projected to triple by 2050, there is an urgent need to integrate OH into dementia care practice‐and‐research (DCPaR).

**Perspective:**

Given that there is currently no cure for AD/ADRD, the importance of prevention and early intervention is heightened, yet oral health—despite its crucial role in systemic health and cognitive function—remains largely overlooked in DCPaR. Chronic oral infections and oral microbiome dysbiosis are significant contributors to systemic inflammation, potentially accelerating AD/ADRD progression. Conversely, cognitive decline impairs individuals’ ability to maintain OH, perpetuating a cycle of worsening health. The Health and Aging Brain Study‐Health Disparities (HABS‐HD) is the most AD/ADRD comprehensive study among diverse communities within the US. While it provides critical insights into aging and cognitive health disparities, it currently lacks direct OH measures. This limitation presents an opportunity to explore OH's impact on cognitive decline and overall quality of life. The Health Equity Scholars Program (HESP) addresses this gap by fostering a diverse, culturally competent workforce to study and treat AD/ADRD. Through the project “Exploring Neurological and Indirect Biomarkers for Potential Correlations with Oral Health: An Interdisciplinary Approach,” HESP and HABS‐HD are advancing efforts to integrate OH into DCPaR, crucial for developing targeted interventions and preventive care for aging populations.

**Call to Action:**

A multidisciplinary approach is essential to address the bidirectional relationship between oral and cognitive health, promote early detection of oral diseases, and implement preventive measures. Incorporating OH into DCPaR aligns with broader health equity goals, addressing disparities in access to care and bridging existing gaps.

**Conclusion:**

As the global burden of AD/ADRD continues to grow, integrating OH into DCPaR will enhance understanding of its role in AD/ADRD progression, improve outcomes for those with cognitive decline, and foster equitable, holistic geriatric healthcare systems.